# Mushroom intake and cognitive performance among US older adults: the National Health and Nutrition Examination Survey, 2011–2014

**DOI:** 10.1017/S0007114521005195

**Published:** 2022-12-14

**Authors:** Djibril M. Ba, Xiang Gao, Laila Al-Shaar, Joshua Muscat, Vernon M. Chinchilli, Paddy Ssentongo, Robert B. Beelman, John Richie

**Affiliations:** 1 Department of Public Health Sciences, Penn State College of Medicine, Hershey, PA, USA; 2 Department of Nutritional Sciences, Penn State University, State College, PA, USA; 3 Department of Food Science and Center for Plant and Mushroom Foods for Health, College of Agricultural Sciences, Pennsylvania State University, University Park, PA, USA

**Keywords:** National Health and Nutrition Examination Survey, Mushroom intake, Cognitive functioning, Epidemiology

## Abstract

Emerging evidence has suggested that mushrooms, which are a rich source of the potent antioxidants ergothioneine and glutathione as well as vitamin D, may have neuroprotective properties. This study investigated the association between mushroom consumption and cognitive performance in a nationally representative sample of US older adults. We analysed data from older adults aged ≥ 60 years from the 2011–2014 National Health and Nutrition Examination Survey. Mushroom intake was measured using up to two 24-h dietary recalls and was categorised into three groups (lowest, middle and highest). Cognitive function tests included the Animal Fluency (AF) Test; Consortium to Establish a Registry for Alzheimer’s Disease Delayed Recall (CERAD-DR) and Word Learning (CERAD-WL); and Digit Symbol Substitution Test (DSST). Multivariable linear regression models were developed, adjusting for socio-demographics, major lifestyle factors, self-reported chronic diseases and dietary factors, including the Healthy Eating Index-2015 score and total energy. The study included 2840 participants. Compared with the lowest category of mushroom intake, participants in the highest category (median intake = 13·4 g /4184 KJ (1000 kcal)/d) had higher scores for DSST (*β* = 3·87; 95 % CI 0·30, 7·45; *P* for trend = 0·03) and CERAD-WL (*β* = 1·05; 95 % CI 0·0003, 2·10; *P* for trend = 0·04). Similar non-significant trends were observed for AF (*β* = 0·24; 95 % CI −2·26, 2·73; *P* for trend = 0·92) but not for the CERAD-DR. Greater mushroom intake was associated with certain cognitive performance tests, suggesting regular mushroom consumption may reduce the risk of cognitive decline.

Chronic diseases, including neurodegenerative diseases, are leading causes of mortality and morbidity in the United States (US) and are associated with modifiable risk factors^(1,2)^. Subjective Cognitive Decline, which is defined as a self-reported acquaintance of worsening or more frequent confusion or memory loss, has emerged as a growing public health issue for the American’s ageing population^(3,4)^. According to the Centers for Disease Control and Prevention, the prevalence of Subjective Cognitive Decline in the US is approximately 11·1 % in adults^(5)^. The process from cognitive decline to dementia is continuous and irreversible, and there is still no effective treatment for dementia^(6)^. It is projected the total number of people with Alzheimer’s disease dementia in the US will reach 13·8 million in 2050 unless preventive measures are developed^(7)^. Available drugs on the market have limited therapeutic value and are associated with various side effects and health complications^(6)^. Thus, there is a need for novel effective preventive methods, including dietary approaches, to lower the risk of developing cognitive impairment. Dietary approaches to prevention are particularly attractive, and findings from two previous longitudinal studies indicated that greater consumption of vegetables was associated with less cognitive decline among older adults^(8,9)^.

Mushrooms are a low-energy, rich source of fiber, vitamins (e.g. B_1_, B_2_, B_12_, vitamin D) and minerals (e.g. Se and Cu)^(10)^. Although mushrooms are considered vegetables, technically, they are not plants but rather belong to the fungi kingdom. They are rich sources of specific bioactive compounds such as antioxidants ergothioneine and glutathione, which protects against cellular oxidative stress, and beneficial carbohydrates including the fibre-associated monosaccharides, chitin and *β*-glucans^(10)^. Oxidative stress plays a significant role in neurodegeneration processes following cognitive impairment and Alzheimer’s disease^(11)^. A previous cross-sectional study suggested that diets high in antioxidants may potentially improve middle-aged adults’ cognitive performance, which could delay the onset of Alzheimer’s disease^(11)^.

Epidemiological findings from our group and others suggested that mushroom consumption is associated with a lower risk of chronic diseases, including some cancers^(12)^, depression,^(13)^ the metabolic syndromes^(14)^, cognitive impairment^(15,16)^ and dementia^(17)^. A study conducted in Norway found that high mushroom intake in the elderly was associated with better cognitive performance than those with very low or no intake^(18)^. However, the association between mushroom consumption and cognitive performance is limited and not well understood. Data on cognitive performance in adults aged 60 years and older were collected from the National Health and Nutrition Examination Survey (NHANES) 2011–2014. Thus, the present study used two cycles (2011–2012, 2013–2014) data from the continuous NHANES. Therefore, we aim to investigate the associations between mushroom consumption and cognitive performance among the US older adults using 2011–2014 data from the NHANES, a nationally representative, cross-sectional survey.

## Methods

### Data source

The NHANES is a series of cross-sectional surveys conducted continually by the National Center for Health Statistics of the Centers for Disease Control and Prevention. The programme is designed to examine adults’ and children’s health and nutritional status of a representative sample of the civilian, non-institutionalised US population^(19)^. The NHANES uses a complex, stratified, multistage probability cluster sampling to select samples located in counties across the US. More details regarding the design and operation of the NHANES have been described elsewhere^(19,20)^. The National Center for Health Statistics Research Ethics Review Board approved the NHANES survey procedures and protocols, and all participants provided written informed consent^(21)^. Detailed information about the dietary interview portion has been published previously^(22)^. Since this study used publicly available de-identified data, an additional Institutional Review Board approval was unnecessary. The current study used two cycles (2011–2012, 2013–2014) data from the continuous NHANES.

### Study population

The current study included participants aged ≥ 60 years who provided at least one reliable and complete 24-h dietary recall data from NHANES 2011–2014 and completed ≥ 1 of 4 cognitive assessments (*n* 2904). As done in a previous study^(23)^, we excluded participants who reported implausible daily energy intake levels (< 3347 KJ (800 kcal) or > 17573 KJ (4200 kcal) for men and < 2092 KJ (500 kcal) or > 14644 KJ (3500 kcal) for women) (*n* 64), leaving a total of 2840 participants.

### Mushroom consumption assessment

Starting in 2003, all examined NHANES participants were eligible for up to two 24-h dietary recall interviews in which respondents reported all foods and beverages consumed during the preceding 24 h. The first day (Day 1) dietary recall interview was conducted in-person in the Mobile Examination Center of NHANES by trained interviewers. The second day (Day 2) dietary recall interview was conducted by telephone 3–10 d after the Mobile Examination Center interviews. Data from 24-h dietary recalls were collected using the US Department of Agriculture (USDA) Automated Multiple-Pass Method to account for day-to-day variation^(19)^.

The USDA Food and Nutrient Databases for Dietary Studies was used to determine the nutrient content of foods. As done by previous studies^(24,25)^, mushroom consumption was reported during NHANES as g/d and determined based on the intake of the USDA food codes, including foods that were mainly mushrooms or mushrooms alone (e.g. egg omelette or scrambled eggs, or dish that was mainly mushrooms, such as mushroom soup, and gravy mushrooms).

The US Environmental Protection Agency-USDA Food Commodity Intake Database commodity codes were used to separate out mushroom in mixed dishes and determine the absolute amounts of mushroom intake as follows: grams of intake by USDA food code times the commodity weight of mushroom contribution per 100 g of the USDA food code^(24)^. Detailed information regarding the Food Commodity Intake Database is described elsewhere^(26)^. Mushroom consumers were defined as those participants who reported eating any amount of mushrooms during the 24-h dietary recalls. We used a 2-d average for participants who completed both recall days and the in-person recall for participants who only completed a single recall. Only participants with reliable and complete dietary records as determined by National Center for Health Statistics were included in the present analysis.

### Cognitive performance assessment

Based on expert input on cognition, a series of assessments for cognitive functioning was introduced during NHANES 2011–2014 surveys allowing for a robust assessment of participants’ cognitive performance^(27)^. Cognitive functioning has been measured periodically in NHANES surveys, either during the household interview or as a component in the Mobile Examination Center. The assessment tests include Consortium to Establish a Registry for Alzheimer’s Disease Delayed Recall (CERAD-DR) and Word Learning (CERAD-WL), the Animal Fluency (AF) and Digit Symbol Substitution Test (DSST). The CERAD test assesses new verbal learning and both immediate and delayed memory^(28,29)^.

The CERAD-WL module consisted of three consecutive word list learning trials of ten words each. Participants were instructed to read words aloud from a computer screen. Immediately following the word learning trials, participants recalled as many words as they could. The CERAD-DR of all ten words was done after the AF test and DSST. For the CERAD-DR test, participants were instructed to recall the ten unrelated words used in the initial CERAD-WL trials. The possible scores on each trial ranged from 0 to 10.

The AF test examines verbal semantic fluency, a component of executive function^(28,30)^. During the AF test, participants were asked to name as many animals as possible in 1 min. A point is given for each named animal. A pretest was done by requiring participants to name three articles of clothing. Participants who were unable to correctly name the three articles of clothing were not eligible to continue with the AF test.

The DSST, a Wechsler Adult Intelligence Scale performance module, was used to evaluate attention and processing speed^(28,31)^. The exercise is conducted using a paper with a key at the top containing nine numbers paired with symbols. Each participant has 2 min to copy the corresponding symbols in the 133 boxes that adjoin the numbers. Participants who were unable to correctly match the symbols with the number during the pretest were not eligible to continue with the main DSST. The score is the total number of correct matches. Detailed information about these assessments has been described previously^(27)^.

Each cognitive test score was primarily analysed in their original continuous scale. In addition, we created a low cognitive performance variable for each test to differentiate low cognitive performance from healthy cognitive function. Presently, there is no gold standard cutoff point to assess low cognitive performance using the CERAD-WL, CERAD-DR, AF and DSST tests. Thus, as done by recently published studies using the same NHANES data, low cognitive performance for the present study was defined using the cutoff scores of < 14 for AF, < 34 for DSST, *<* 17 for CERAD-WL and *<* 5 for CERAD-DR^(32,33)^.

### Covariates assessment

The following covariates were included in our analysis: age (years), sex (men/women), ethnicity-race (Mexican American, other Hispanic, Non-Hispanic White, other race-multi-racial), education (less than high school, high school degree, more than high school), family poverty to income ratio (≤ 1·30, >1·30), smoking status (non-smoker, former smoker, current smoker), physical activity MET-min/week (< 600, 600–1199, ≥ 1200), alcohol intake (g/d), total energy intake KJ (kcal)/d, history of CVD, history of diabetes and the Healthy Eating Index-2015 (HEI-2015) score, a measure of diet quality with a higher score indicating better diet quality. Because individuals 80 and over are top-coded at 80 years of age in the NHANES in order to protect participants’ confidentiality, thus age groupings were created as follows: age (60 to < 70 years, 70 to < 80 years and ≥ 80 years). These covariates were selected because they have shown to be associated with both cognitive functioning and mushroom intake^(13,15,25)^. A previous study indicated that mushroom consumers in the US were more likely to be non-Hispanic White^(34)^. In addition, older African Americans are more likely to score low on a wide range of cognitive assessments than non-Hispanic Whites^(35,36)^. Another previous study indicated that greater household income was associated with higher scores across all cognitive domains^(37)^. Furthermore, diabetes has been related to both mushroom consumption^(38)^ and cognitive impairment^(39)^. The HEI-2015 shares the same components as the HEI-2010, except saturated fat and added sugars replaced empty calories, resulting in thirteen components^(40)^. The thirteen components of the HEI-2015 include total fruit, whole fruit, total vegetables, greens and beans, whole grains, dairy, total protein foods, seafood and plant proteins, fatty acids, refined grains, sodium, added sugars and saturated fats^(40)^. The HEI-2015 total score ranges from 0 to 100 and was calculated using the Food Pattern Equivalents Database and publicly available SAS macro code from the National Cancer Institute website (https://epi.grants.cancer.gov/hei/sas-code.html). Physical activity was assessed via self-reported using the Global Physical Activity Questionnaire^(41)^. Measures of physical activity were calculated based on the three domains in which physical activity is performed, such as leisure-time physical activity, transportation-related physical activity and domestic physical activity. A total physical activity score metabolic equivalents of task (MET)-min/week was calculated by summing the total MET-min from each domain. The total score MET-min/week was categorised into three groups (< 600, 600–1199 and ≥ 1200)^(42)^. History of CVD and diabetes were defined as self-reported physician diagnosis of CVD and diabetes.

### Post-hoc power analysis and sample size

This study had at least 90 % power to detect significant regression coefficients of 3·87 (DSST), 1·05 (CERAD-WL), 0·21 (CERAD-DR) and 0·24 (AF), between each of the cognitive test scores and mushroom intake, assuming (a) sd of 17·26 (for DSST), 4·89 (for CERAD-WL), 2·37 (for CERAD-DR), 5·52 (for AF) and 3·47 (for mushroom intake), (b) sample size of 2789 (*n* 2737 in the lowest category *v*. 52 in the highest category of mushroom intake), (c) type 1 error of 5 % and (d) a null hypothesis of *β*-coefficients equal to zero.

### Statistical analysis

All statistical analyses were performed using SAS statistical software version 9.4 (SAS Institute). Consistent with National Center for Health Statistics guidelines for analysing NHANES data, all analyses were conducted using appropriate sampling weights, clustering and stratification to account for the complex sampling design^(19)^.

Participants’ characteristics were presented as means and standard errors for continuous variables or proportions for categorical variables. Multivariable linear regression models (proc surveyreg; SAS institute) with appropriate sampling weight, cluster and strata were used to examine the association of mushroom consumption with each cognitive function in its original continuous scale (CERAD-WL, CERAD-DR, AF and DSST scores), adjusting for potential confounders including age, sex, ethnicity-race, education, family poverty to income ratio, smoking status, physical activity, alcohol intake, total energy intake, history of CVD, history of diabetes and the HEI-2015 score. We also applied multivariable logistic regression models using the SAS surveylogistic procedure to examine the association of mushroom consumption with low cognitive function using the cutoff scores of < 14 for AF, < 34 for DSST, *<* 17 for CERAD-WL and *<* 5 for CERAD-DR^(32,33)^ adjusting for the same covariates mentioned above. Imputation was performed for participants with missing demographics, lifestyle and self-reported diseases variables using the fully conditional specification method^(43)^. Variables’ distributions were checked before and after imputation, and the data structure showed to be well preserved. We used SAS Rank procedure to categorise mushroom intake into three categories: lowest (0 g/4184 KJ (1000 kcal)/d, *n* 2737), middle (median intake = 2·4 g/4184 KJ (1000 kcal)/d, range = 5·1, *n* 51) and highest (median intake = 13·4 g/4184 KJ (1000 kcal)/d, range = 92·4, *n* 52). As done in previous studies, the multivariable surveyreg results were presented as *β*-coefficient with 95 % CI, and multivariable surveylogistic regression results were presented as adjusted OR with 95 % CI. Trends across groups were assessed using the median values of each group as a single continuous variable. All *P* values were two-sided.

## Results

Table 1 presents the characteristics of the 2840 study participants by mushroom intake status. More than half (56·0 %) were aged 60–69 years, and 53·4 % were women. Nearly 77·0 % were non-Hispanic White, and 61·5 % had more than a high school degree. The weighted prevalence of mushroom intake was 4·2 % (95 % CI 2·9, 5·5). Participants with higher mushroom intake were more likely to be non-Hispanic White than those with the no intake group (Table 1).

**Table 1.** Baseline characteristics of the study participants aged ≥ 60 years, National Health and Nutrition Examination Survey (NHANES) 2011–2014 (Numbers and percentages; mean values with their standard error of the mean, *n* 2840)*

	Mushroom intake					
	Lowest (*n* 2737)		Middle (*n* 51)		Highest (*n* 52)	
Characteristic	*n*	%	*n*	%	*n*	%
g/4184 KJ (1000 kcal)/d						
Median	0		2·4		13·4	
Range			5·1		92·4	
Age groups						
60–69	1464	56·5	26	41·6	24	48·5
70–79	816	28·8	19	40·8	14	31·6
80 and older	457	14·7	6	17·6	14	19·8
Women	1405	53·0	27	60·4	29	63·2
Non-Hispanic white	1297	76·7	33	89·4	33	86·1
Education above high school	1372	61·3	29	54·9	36	73·2
Family PIR ≤ 1·30	849	19·3	13	19·8	10	12·5
Current smoker	343	10·1	10	13·2	1	7·1
PA (≥ 1200 MET-min/week)	1463	56·6	29	54·6	35	73·6
History of CVD	618	21·8	10	21·0	11	22·2
History of diabetes	783	23·2	10	10·9	11	14·4
Alcohol intake g/d						
Mean	7·1		12·8		5·8	
se	0·6		3·7		1·3	
Total energy KJ (kcal)/d						
Mean	7929 (1895)		8259 (1974)		7046 (1684)	
se	84 (20)		339 (81)		272 (65)	
HEI-2015 score						
Mean	57·9		58·7		56·8	
se	0.6		2.9		3.0	

PA, physical activity; PIR, poverty income ratio; HEI, Healthy Eating Index.

* All Ns are unweighted and all proportions and means (se) are survey-weighted for complex survey design.

Upon analysing each cognitive function score in its original continuous scale (Table 2), compared with the lowest group, participants in the highest group (median intake = 13·4 g /4184 KJ (1000 kcal)/d) had higher scores for DSST (*β* = 3·87; 95 % CI 0·30, 7·45; *P* for trend = 0·03) and CERAD-WL (*β* = 1·05; 95 % CI 0·0003, 2·10; *P* for trend = 0·04). Similar non-significant trends were observed for AF score (*β* = 0·24; 95 % CI −2·26, 2·73; *P* for trend = 0·92).

**Table 2.** Regression coefficients (*β*) and 95 % confidence intervals for cognitive test scores in their original continuous scale by mushroom intake, NHANES 2011–2014 (Coefficients and 95 % confidence intervals)

	Mushroom intake					
		Middle		Highest		
	Lowest	Regression coefficients (*β*)	95 % confidence intervals	Regression coefficients (*β*)	95 % confidence intervals	*P*-trend
Intake, g/4184 KJ (1000 kcal)/d						
Median	0	2·4		13·4		
Range		5·1		92·4		
DSST						
Model 1	1 (reference)	2·27	–3·50, 8·04	5·05	–0·14, 10·24	0·04
Model 2	1 (reference)	0·51	–5·81, 6·83	3·87	0·30, 7·45	0·03
CERAD-WL						
Model 1	1 (reference)	0·78	–0·35, 1·91	1·04	–0·03, 2·11	0·03
Model 2	1 (reference)	0·51	–0·69, 1·70	1·05	0·0003, 2·10	0·04
CERAD-DR						
Model 1	1 (reference)	0·38	–0·35, 1·11	0·25	–0·67, 1·17	0·50
Model 2	1 (reference)	0·26	–0·54, 1·06	0·21	–0·62, 1·04	0·54
AF						
Model 1	1 (reference)	–0·20	–2·84, 2·44	0·40	–2·85, 3·64	0·82
Model 2	1 (reference)	–0·78	–3·74, 2·17	0·24	–2·26, 2·73	0·92

AF, Animal Fluency Test; CERAD-DR, Consortium to Establish a Registry for Alzheimer’s Disease Delayed Recall test; CERAD-WL, Consortium to Establish a Registry for Alzheimer’s Disease Word Learning test; DSST, Digit Symbol Substitution Test.

Model 1: Age, sex, education adjusted.

Model 2: Model 1 + further adjustment of ethnicity-race, ratio of family income to poverty level, smoking status, physical activity, history of diabetes, history of CVD, total energy, alcohol intake, HEI-2015 score.

In the multivariable logistic regression analysis (Table 3) compared with the lowest group, those in the highest group of mushroom intake had lower odds of scoring low on the DSST (adjusted OR = 0·29; 95 % CI 0·11, 0·76; *P* for trend = 0·06) and on the CERAD-WL (adjusted OR = 0·43; 95 % CI 0·18, 0·99; *P* for trend = 0·04). Similar trends were also observed with AF, but these associations were not significant. Unexpectedly, we found that compared with the lowest group, those in the middle group had lower odds of scoring low on the CERAD-DR (adjusted OR = 0·36; 95 % CI 0·14, 0·95) but not for the highest group (adjusted OR = 1·41; 95 % CI 0·58, 3·38) (*P* for trend = 0·61).

**Table 3.** Adjusted odds ratio (95 % confidence intervals) in relation to the odds of a low cognitive score by mushroom intake, NHANES 2011–2014 (Odds ratios and 95 % confidence intervals)

	Mushroom intake					
		Middle		Highest		
	Lowest	Odds ratio	95 % confidence intervals	Odds ratio	95 % confidence intervals	*P*-trend
Intake, g/4184 KJ (1000 kcal)/d						
Median	0	2·4		13·4		
Range		5·1		92·4		
DSST < 34						
No. of cases	661	9		5		
Model 1	1 (reference)	0·91	0·24, 3·41	0·21	0·07, 0·61	0·01
Model 2	1 (reference)	1·33	0·28, 6·23	0·29	0·11, 0·76	0·06
CERAD-WL < 17						
No. of cases	819	10		12		
Model 1	1 (reference)	0·63	0·21, 1·92	0·40	0·20, 0·84	0·01
Model 2	1 (reference)	0·69	0·24, 2·02	0·43	0·18, 0·99	0·04
CERAD-DR < 5						
No. of cases	705	10		15		
Model 1	1 (reference)	0·34	0·13, 0·91	1·25	0·52, 2·98	0·82
Model 2	1 (reference)	0·36	0·14, 0·95	1·41	0·58, 3·38	0·61
AF < 14						
No. of cases	831	12		13		
Model 1	1 (reference)	0·90	0·29, 2·77	0·73	0·28, 1·90	0·89
Model 2	1 (reference)	1·17	0·33, 4·12	0·74	0·30, 1·84	0·56

AF, Animal Fluency Test; CERAD-DR, Consortium to Establish a Registry for Alzheimer’s Disease Delayed Recall test; CERAD-WL, Consortium to Establish a Registry for Alzheimer’s Disease Word Learning test; DSST, Digit Symbol Substitution Test.

Model 1: Age, sex, education adjusted.

Model 2: Model 1 + further adjustment of ethnicity-race, ratio of family income to poverty level, smoking status, physical activity, history of diabetes, history of CVD, total energy, alcohol intake, HEI-2015 score.

## Discussion

In this study using continuous NHANES data of US older adults, we found that the weighted prevalence of participants who reported consuming mushrooms in their past 24-h recall dietary data was only 4·2 % (95 % CI 2·9, 5·5). In the multivariable linear regression analyses, we found that compared with the lowest category of mushroom intake, participants in the highest category had higher scores for DSST and CERAD-WL. In addition, we observed a significant linear trend relationship between greater mushroom intake and cognitive performance for DSST and CERAD-WL but not for CERAD-DR and AF. Consistently, when cognitive function scores were dichotomised to differentiate between low and normal cognitive function, we found that participants with the highest mushroom consumption had lower odds of having a low cognitive performance for DSST and CERAD-WL. In this full adjusted model, we found that the *P* values for linear trend were significant for CERAD-WL and marginally significant for DSST. However, no significant trends were observed between greater mushroom intake and CERAD-DR and AF. The observed associations were independent of socio-demographics, major lifestyle risk factors, self-reported chronic diseases and other dietary factors. Overall, greater mushroom intake was associated with better cognitive performance for DSST and CERAD-WL. The findings suggest that regular mushroom consumption may be protective against cognitive impairment.

Mushroom intake was very low in this study (4·2 %) compared with a previous study where the proportion of mushroom consumers was 25 %^(18)^. In addition, cognitive function assessments used in this study were different from previous studies^(18,44)^. Lastly, dietary data assessed in this study were done using a 24-h dietary recall compared with the FFQ used in previous studies mentioned above. Notwithstanding, our findings are generally consistent with previous studies that have assessed the associations between mushroom consumption and cognitive health. A study conducted by Nurk and colleagues in Norway indicated that individuals with high mushroom intake performed significantly better in cognitive tests compared with those with low or no intake^(18)^. Another prospective cohort study conducted in the Netherlands showed that higher mushroom intake was associated with better cognitive function at baseline^(44)^. A recent community-based cross-sectional study conducted in Singapore showed that people who consumed more than two portions of mushrooms per week had lower odds of mild cognitive impairment^(15)^. The authors of this study suggested that the bioactive compounds in mushrooms have the potential to delay neurodegeneration^(15)^. Another study conducted by Zhang and colleagues indicated that frequent mushroom consumption was significantly associated with a lower risk of incident dementia in elderly Japanese^(17)^.

The association between mushroom consumption and certain cognitive function tests observed in this study is plausible and may stem from bioactive compounds found in mushrooms. However, our findings should be interpreted with caution. We had a small sample size, and cognitive function data in adults aged 60 years and older were available in the NHANES 2011–2014. Thus, we could not add additional NHANES cycles to the present study to improve our sample size.

Mushrooms are an excellent source of a variety of essential dietary micronutrients and contain high amounts of potent antioxidants that can mitigate oxidative stress and improve cognitive health. Indeed, oxidative stress plays a significant role in neurodegeneration processes following cognitive impairment and Alzheimer’s disease^(11)^. A study conducted by Beydoun and colleagues using NHANES data suggested that dietary antioxidant intake was associated with better cognitive performance^(11)^. Of particular importance, mushrooms are uniquely high in the potent antioxidant ergothioneine. This sulphur amino acid is obtained exclusively through dietary sources, with mushrooms having the highest concentrations of any other dietary component^(45–47)^. Ergothioneine has been reported to have neuroprotective properties^(48,49)^.

Our study has major strengths. To the best of our knowledge, this is the first study that used nationally representative US older adult population data and examined the associations between mushroom consumption and cognitive performance. Our study used comprehensive methods to assess cognitive functions. DSST has been shown to provide a practical and effective method to monitor cognitive functions over time in clinical practice^(50,51)^. Our findings are robust to adjustment for a wide spectrum of potential confounders factors, given their potential association with cognitive functions.

When interpreting our findings, several limitations should be considered. First, the diet was assessed using up to two 24-h recalls, which may not have adequately captured the within-person variation in mushroom intake. Such non-differential measurement error may have underestimated the strength of the association of mushroom intake with cognitive performance. Second, the cross-sectional design precludes establishing a clear temporal sequence. Third, we did not have information on the different types of mushrooms species consumed in the NHANES database. Lastly, the NHANES nutrients database does not contain information on ergothioneine and glutathione intake; therefore, we could not assess the associations between these antioxidants found in mushrooms and cognitive performance in this analysis.

### Conclusions

Using nationally representative US older adults, we found that compared with the lowest category of mushroom intake, participants in the highest category had higher scores for DSST and CERAD-WL, suggesting that higher mushroom consumption may reduce the risk of cognitive decline in older adults. Such findings may play an important role in the prevention of neurodegenerative diseases. We used cognitive function assessments data available in the NHANES 2011–2014; thus, we could not add additional NHANES cycles to the current study to improve our sample size. Larger epidemiological studies with repeated diet measurements are warranted to replicate further our findings regarding mushrooms’ potential health benefits and cognitive health.
